# Supplementary material for: Assembly and functionality of the ribosome with tethered subunits
Source: Nat Commun. 2019 Feb 25;10:930. doi: 10.1038/s41467-019-08892-w (PMC6389949; doi:10.1038/s41467-019-08892-w)
Supplement: Supplementary file 1 — Supplementary Information [file 41467_2019_8892_MOESM1_ESM.pdf]

SUPPLEMENTARY INFORMATION

Assembly and functionality of the ribosome with tethered subunits

Nikolay A. Aleksashin, Margus Leppik, Adam J. Hockenberry, Dorota Klepacki, Nora Vázquez-Laslop, Michael C. Jewett, Jaanus Remme and Alexander S. Mankin

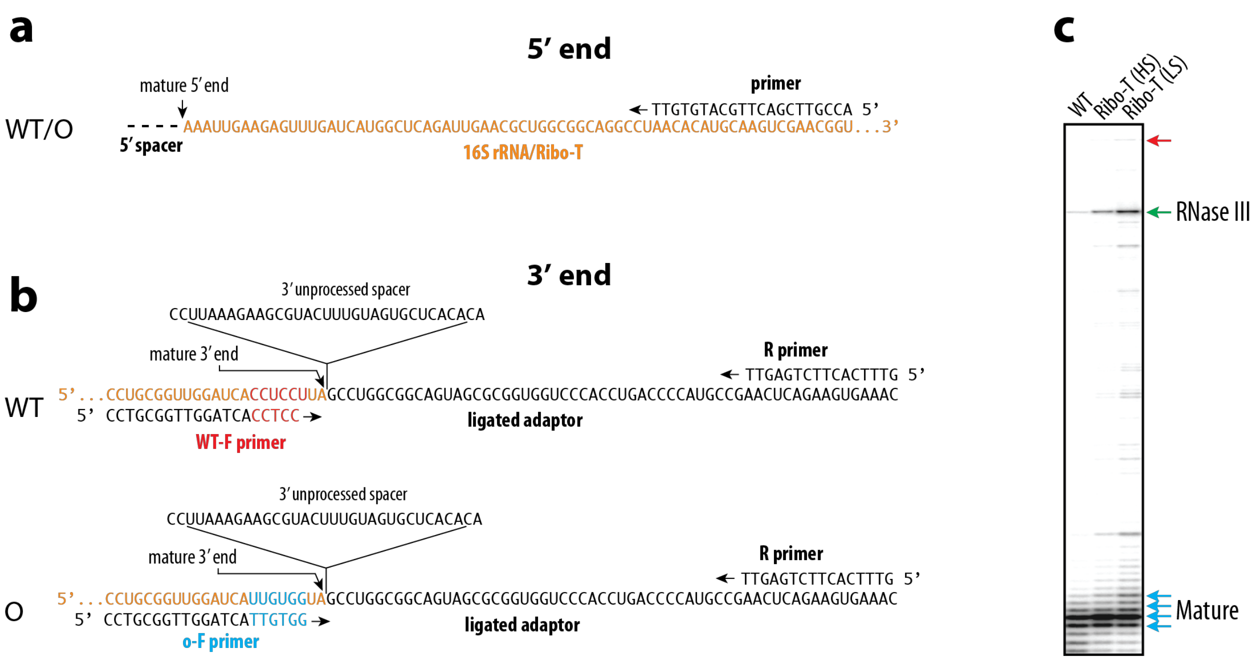

Supplementary Figure 1. Analysis of the Ribo-T rRNA processing. a,b The locations of the primers used for the analysis of the (**a**) 5’ and (**b**) 3’ rRNA processing. Mature rRNA sequences are shown in orange. In (b) wt SD sequence is shown in red and o-ASD sequence is indicated in cyan. **c** Primer extension analysis of the 5’ end of the rRNA isolated from 70S ribosomes (wt), heavy shoulder (HS) or light shoulder (LS) of the Ribo-T peak obtained by sucrose gradient centrifugation (see **Fig. 2a** in the main text). The cDNA bands corresponding to the mature rRNA ends, to the product of RNase III cleavage and to rRNA uncleaved by RNase III are indicated by cyan, green and red arrows, respectively (see **Fig. 4a** in the main text).

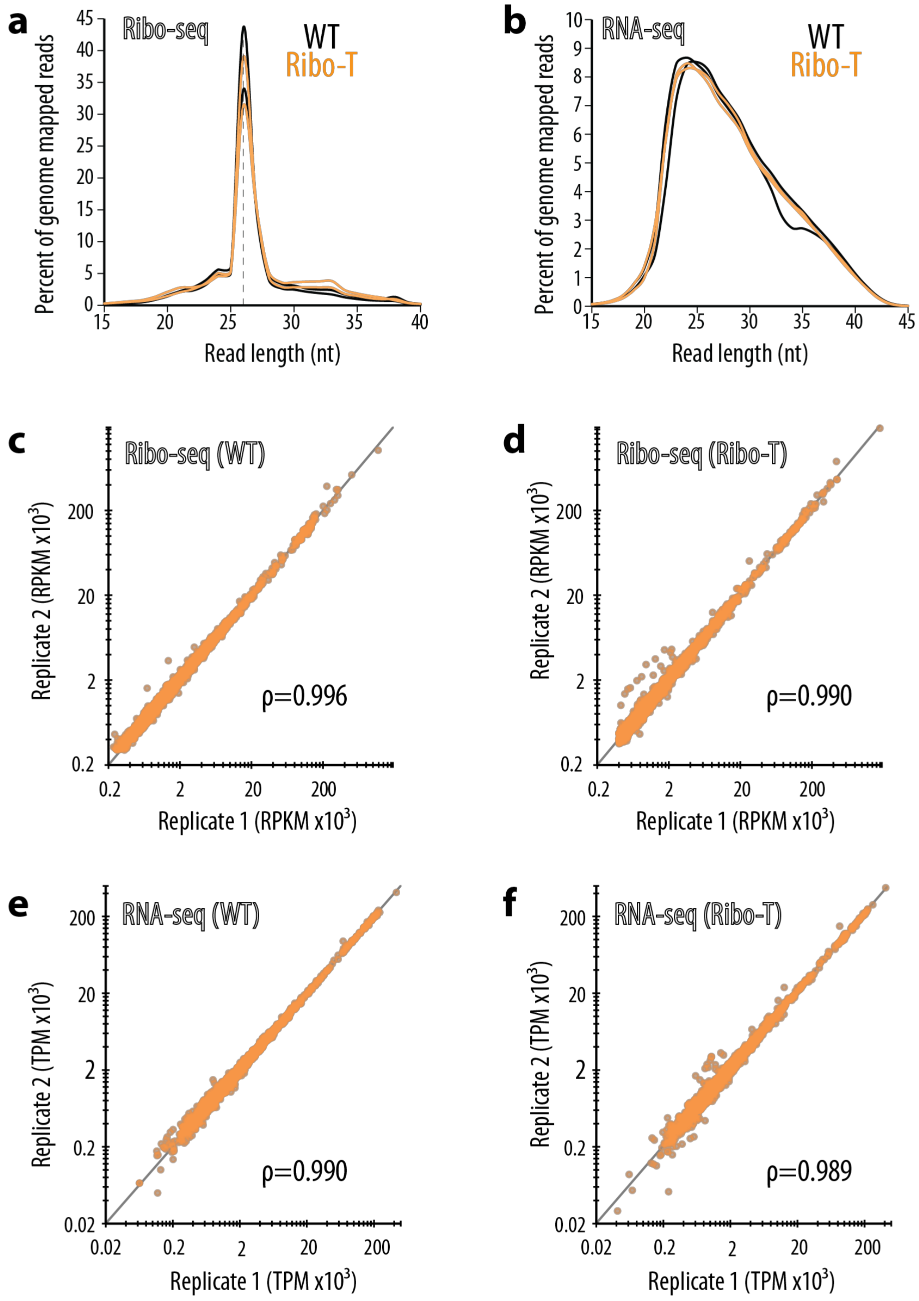

Supplementary Figure 2. Read lengths and reproducibility of the Ribo-seq and RNA-seq experiments. a,b Distribution of lengths of the reads in two Ribo-T samples (orange contours) and two control (wt) samples (black contours) in (**a**) Ribo-seq and (**b**) RNA-seq datasets. **c-f**, Reproducibility of the per-gene read counts comparing biological replicates in (**c,d**) Ribo-seq and (**e,f**) RNA-seq data. The Ribo-seq analysis was carried out on 1596 high-coverage genes. Spearman correlation coefficients are indicated.

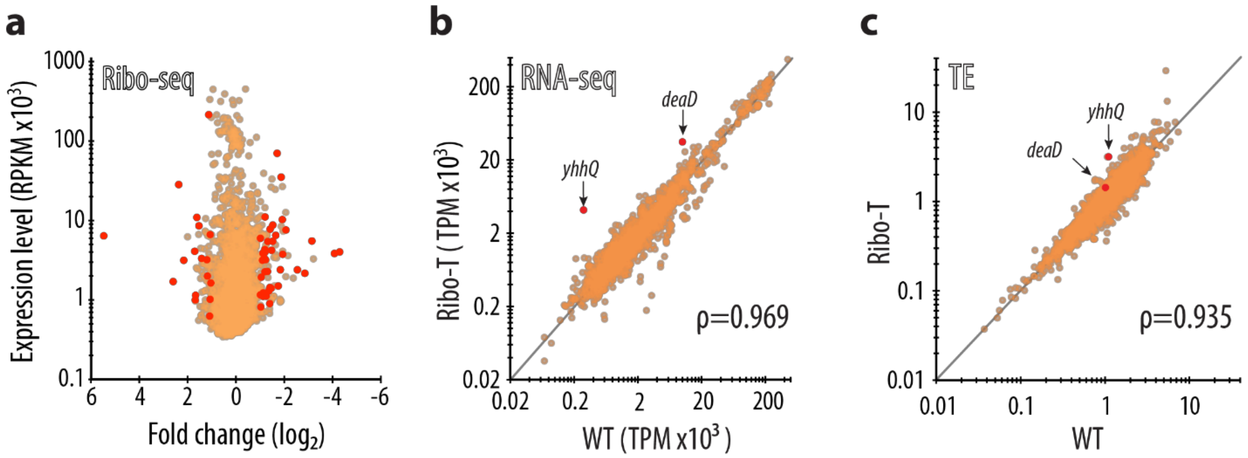

Supplementary Figure 3. Comparison of the translatome, transcriptome and translation efficiency in the Ribo-T and wt cells. a. Changes in gene expression in Ribo-T cells in comparison with control (wt) cells deduced from Ribo-seq data. Each dot represents one of the 1596 high-coverage genes. Expression level was calculated as geometric mean of the number of reads (RPKM) mapped to the gene in four Ribo-seq samples (Ribo-T and wt, two biological replicates for each). Genes characterized with more than 2-fold difference in expression and called statistically significant (Student's t-test, FDR corrected $p < 0.02$) are highlighted in red. **b.** Comparison of average mRNA-seq abundances (transcripts per million, TPM) in Ribo-T and control (wt) cells. **c**. Comparison of average translation efficiency calculated as a ratio between the Ribo-seq (RPKM) and RNA-seq (RPKM) abundances for each gene. In **a, b** and **c**, data are for 1596 high-coverage genes in the Ribo-seq samples. In **b** and **c**, example translation-related genes *yhhQ* and *deaD* are highlighted. Spearman correlation coefficients are indicated.

Supplementary Table 1. Number of reads in Ribo-seq and RNA-seq experiments.

**Sample**	**Sample type**	**N reads after barcode disambiguation**	**N reads mapped to the non-coding RNA genes**	**N reads mapped to the rest of the genome**
WT replicate 1	Ribo-seq	66,713,599	35,406,766	26,082,965
WT replicate 2	Ribo-seq	59,193,645	36,991,128	18,087,601
Ribo-T replicate 1	Ribo-seq	49,933,355	32,182,325	14,172,828
Ribo-T replicate 2	Ribo-seq	47,827,088	27,409,962	16,242,739
WT replicate 1	RNA-seq	30,252,124	23,016,726	5,440,005
WT replicate 2	RNA-seq	29,141,273	22,207,303	5,149,843
Ribo-T replicate 1	RNA-seq	31,566,411	21,492,111	7,615,602
Ribo-T replicate 2	RNA-seq	21,985,102	14,565,024	5,767,715

Supplementary Table 2. Genes with a more than two-fold change in expression level and called statistically significant (Student's T-test, FDR corrected p < 0.02) in Ribo-T cells compared to wt control (from Ribo-seq data).

Gene ID/name	wt average RPKM	Ribo-T average RPKM	fold change Log_2	p-value (FDR-corrected)
**Up-regulated genes**				
b3471/yhhQ	282.34	12543.24	5.47	0.0126
b1112/bhsA	482.61	2924.64	2.60	0.0121
b3162/deaD	9190.24	47489.00	2.37	0.0190
b0607/uspG	1153.11	5156.10	2.16	0.0167
b3183/obgE	1924.30	6280.85	1.71	0.0144
b1376/uspF	474.22	1524.50	1.68	0.0167
b1635/gstA	547.22	1741.28	1.67	0.0144
b4314/fimA	5377.90	16505.64	1.62	0.0144
b1446/ydcY	4408.59	12752.47	1.53	0.0121
b4179/rnr	1809.60	4848.16	1.42	0.0159
b4595/yciY	1941.32	4470.45	1.20	0.0148
b2017/yefM	1224.06	2775.32	1.18	0.0144
b1089/rpmF	134997.14	294455.66	1.13	0.0165
b1708/nlpC	400.35	852.12	1.09	0.0154
b0199/metN	657.87	1382.15	1.07	0.0121
b2193/narP	4305.79	9005.80	1.06	0.0121
b1245/oppC	1070.98	2203.08	1.04	0.0121
**Down-regulated genes**				
b3433/asd	1549.93	768.56	-1.01	0.0121
b2551/glyA	7997.66	3959.31	-1.01	0.0174
b4322/uxuA	1093.78	535.66	-1.03	0.0121
b0720/gltA	2609.78	1262.92	-1.05	0.0157
b2114/metG	4304.76	2014.23	-1.10	0.0121
b3401/hslO	5305.70	2458.33	-1.11	0.0121
b1914/uvrY	1525.34	698.71	-1.13	0.0144
b0767/pgl	1689.58	772.03	-1.13	0.0189
b2094/gatA	4660.43	2069.19	-1.17	0.0121

b2597/raiA	6016.73	2624.43	-1.20	0.0167
b1062/pyrC	3149.30	1373.41	-1.20	0.0121
b0970/yccA	5584.25	2425.26	-1.20	0.0159
b0411/tsx	15465.66	6711.11	-1.20	0.0121
b3616/tdh	4486.71	1919.23	-1.23	0.0121
b2143/cdd	1571.50	661.04	-1.25	0.0165
b4381/deoC	3250.55	1315.52	-1.31	0.0121
b0154/hemL	7774.69	3122.56	-1.32	0.0144
b3498/prlC	1869.84	728.31	-1.36	0.0121
b0759/galE	1306.73	495.26	-1.40	0.0159
b3408/feoA	2087.32	787.05	-1.41	0.0159
b1101/ptsG	11308.55	4246.11	-1.41	0.0165
b0945/pyrD	2050.51	747.63	-1.46	0.0165
b0237/pepD	6209.35	2229.16	-1.48	0.0144
b4025/pgi	8127.16	2825.15	-1.52	0.0158
b2096/gatY	13012.65	4517.58	-1.53	0.0121
b3919/tpiA	9884.99	3154.96	-1.65	0.0121
b1779/gapA	107378.81	32943.23	-1.70	0.0140
b0459/maa	2314.94	688.29	-1.75	0.0189
b2091/gatD	3753.96	1049.19	-1.84	0.0176
b3908/sodA	55252.08	15098.26	-1.87	0.0144
b0032/carA	16241.35	4291.88	-1.92	0.0189
b3617/kbl	5958.34	1558.35	-1.93	0.0144
b1817/manX	12272.53	2928.00	-2.07	0.0144
b0150/fhuA	4090.63	702.48	-2.54	0.0144
b4139/aspA	3803.30	526.76	-2.85	0.0154
b1452/yncE	9930.12	1123.01	-3.14	0.0121
b4244/pyrI	7258.04	429.81	-4.08	0.0144
b4245/pyrB	7656.84	390.48	-4.29	0.0127

Supplementary Table 3. Plasmids used in this study.

Plasmid name	Description
pAM552[1]	Carries *E. coli rrnB* operon under control of the P_L promoter
poAM552[*]	Same as pAM552 but ASD (CCUCCU) in 16S rRNA is changed to oASD (UUGUGG)
pRibo-T[1]	Carries Ribo-T operon under control of the P_L promoter
poRibo-T[*]	Same as pRibo-T but ASD is changed to oASD

* Provided by E. Carlson, Northwetsern University

Supplementary references

1. Orelle, C. et al. Protein synthesis by ribosomes with tethered subunits. *Nature* **524**, 119-124 (2015).
