# Supplementary material for: Assembly and functionality of the ribosome with tethered subunits
Source: Nat Commun. 2019 Feb 25;10:930. doi: 10.1038/s41467-019-08892-w (PMC6389949; doi:10.1038/s41467-019-08892-w)
Supplement: Supplementary file 3 — Description of Additional Supplementary Files [file 41467_2019_8892_MOESM3_ESM.pdf]

Title: Supplementary Data 1
Description: Ribo-seq master file

Title: Supplementary Data 2
Description: RNA-seq master file
